# A Rare Case of Duodenal Pseudomelanosis

**DOI:** 10.3390/diagnostics11112152

**Published:** 2021-11-20

**Authors:** Marianna D’Ercole, Gianluca Lopez, Luca Elli, Stefano Ferrero, Giorgio Alberto Croci

**Affiliations:** 1 Pathology Unit, Fondazione IRCCS Ca’ Granda Ospedale Maggiore Policlinico, 20122 Milan, Italy; marianna.dercole@unimi.it (M.D.); stefano.ferrero@unimi.it (S.F.); giorgio.croci@unimi.it (G.A.C.); 2 School of Pathology, University of Milan, 20122 Milan, Italy; 3 Gastroenterology Unit, Fondazione IRCCS Ca’ Granda Ospedale Maggiore Policlinico, 20122 Milan, Italy; luca.elli@policlinico.mi.it; 4 Department of Biomedical, Surgical and Dental Sciences, University of Milan, 20122 Milan, Italy; 5 Department of Pathophysiology and Transplantation, University of Milan, 20122 Milan, Italy

**Keywords:** pseudomelanosis, duodenal, duodeni, pigmentation, iron, endoscopy

## Abstract

A black-spotted duodenal mucosa was observed during endoscopy of a man with several comorbidities including hypertension and end-stage kidney disease. Histopathological examination revealed pigment-laden macrophages in the lamina propria of the duodenal villi, which was consistent with duodenal pseudomelanosis.

**Figure 1:**
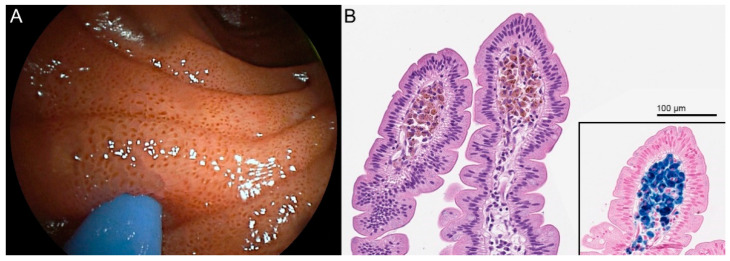
A 75-year-old man underwent endoscopy for obstructive lithiasic cholangitis. His medical history included gastric resection for non-Hodgkin lymphoma, monoclonal gammopathy of undetermined significance (MGUS), HCV infection, hypertension, and stage 4 chronic kidney disease. His medications included furosemide, metoprolol, and amlodipine. During the endoscopy, the duodenal mucosa presented spotted black pigmentation at the tip of the villi (**A**). Duodenal biopsy samples stained with routine hematoxylin and eosin (**B**) showed aggregates of pigment-laden macrophages in the lamina propria of the apical portion of the villi, which tested intensely positive with Perl’s stain for iron; enterocytes demonstrated a faint positivity for Perl’s Prussian blue underneath the microvilli (**B**, inset). These findings were consistent for duodenal pseudomelanosis, a benign condition which harbors no known clinical sequelae [1,2,3,4,5,6,7,8,9,10,11,12,13].

## Data Availability

Not applicable.
